# Role of female-predominant MYB39-bHLH13 complex in sexually dimorphic accumulation of taxol in *Taxus media*

**DOI:** 10.1093/hr/uhac062

**Published:** 2022-03-14

**Authors:** Chunna Yu, Jiefang Huang, Qicong Wu, Chengchao Zhang, Xiao-lin Li, Xinyun Xu, Shangguo Feng, Xiaori Zhan, Zhehao Chen, Huizhong Wang, Chenjia Shen

**Affiliations:** College of Life and Environmental Sciences, Hangzhou Normal University, Hangzhou 310036, China; Zhejiang Provincial Key Laboratory for Genetic Improvement and Quality Control of Medicinal Plants, Hangzhou Normal University, Hangzhou 310036, China; College of Life and Environmental Sciences, Hangzhou Normal University, Hangzhou 310036, China; Zhejiang Provincial Key Laboratory for Genetic Improvement and Quality Control of Medicinal Plants, Hangzhou Normal University, Hangzhou 310036, China; College of Life and Environmental Sciences, Hangzhou Normal University, Hangzhou 310036, China; Zhejiang Provincial Key Laboratory for Genetic Improvement and Quality Control of Medicinal Plants, Hangzhou Normal University, Hangzhou 310036, China; College of Life and Environmental Sciences, Hangzhou Normal University, Hangzhou 310036, China; Zhejiang Provincial Key Laboratory for Genetic Improvement and Quality Control of Medicinal Plants, Hangzhou Normal University, Hangzhou 310036, China; State Key Laboratory Breeding Base of Dao-di Herbs, National Resource Center for Chinese Materia Medica, China Academy of Chinese Medical Sciences, Beijing 100700, China; College of Life and Environmental Sciences, Hangzhou Normal University, Hangzhou 310036, China; Zhejiang Provincial Key Laboratory for Genetic Improvement and Quality Control of Medicinal Plants, Hangzhou Normal University, Hangzhou 310036, China; College of Life and Environmental Sciences, Hangzhou Normal University, Hangzhou 310036, China; Zhejiang Provincial Key Laboratory for Genetic Improvement and Quality Control of Medicinal Plants, Hangzhou Normal University, Hangzhou 310036, China; College of Life and Environmental Sciences, Hangzhou Normal University, Hangzhou 310036, China; Zhejiang Provincial Key Laboratory for Genetic Improvement and Quality Control of Medicinal Plants, Hangzhou Normal University, Hangzhou 310036, China; College of Life and Environmental Sciences, Hangzhou Normal University, Hangzhou 310036, China; College of Life and Environmental Sciences, Hangzhou Normal University, Hangzhou 310036, China; Zhejiang Provincial Key Laboratory for Genetic Improvement and Quality Control of Medicinal Plants, Hangzhou Normal University, Hangzhou 310036, China; College of Life and Environmental Sciences, Hangzhou Normal University, Hangzhou 310036, China; Zhejiang Provincial Key Laboratory for Genetic Improvement and Quality Control of Medicinal Plants, Hangzhou Normal University, Hangzhou 310036, China

## Abstract

*Taxus* trees are major natural sources for the extraction of taxol, an anti-cancer agent used worldwide. *Taxus media* is a dioecious woody tree with high taxol yield. However, the sexually dimorphic accumulation of taxoids in *T. media* is largely unknown. Our study revealed high accumulation of taxoids in female *T. media* trees using a UPLC–MS/MS method. Thereafter, many differential metabolites and genes between female and male *T. media* trees were identified using metabolomic and transcriptomic analyses, respectively. Most of the taxol-related genes were predominantly expressed in female trees. A female-specific R2R3-MYB transcription factor gene, *TmMYB39*, was identified. Furthermore, bimolecular fluorescence complementation and yeast two-hybrid assays suggested the potential interaction between TmMYB39 and TmbHLH13. Several taxol biosynthesis-related promoter sequences were isolated and used for the screening of MYB recognition elements. The electrophoretic mobility shift assay indicated that TmMYB39 could bind to the promoters of the *GGPPS*, *T10OH*, *T13OH*, and *TBT* genes. Interaction between TmMYB39 and TmbHLH13 transactivated the expression of the *GGPPS* and *T10OH* genes. TmMYB39 might function in the transcriptional regulation of taxol biosynthesis through an MYB-bHLH module. Our results give a potential explanation for the sexually dimorphic biosynthesis of taxol in *T. media*.

## Introduction

Trees of the genus *Taxus* (yews) are fundamental natural sources for the extraction of taxol, one of the most important anti-cancer agents [1]. Paclitaxel (also named taxol) has been frequently used for the treatment of different specific cancers, such as ovarian and metastatic breast cancers. With the increase in cancer risk, the market demand for taxol is also increasing. The high commercial value of taxol has resulted in considerable denudation and extermination of wild *Taxus* trees in the recent years [2].

To obtain full understanding of taxol biosynthesis, generous studies have been performed to decipher its biosynthesis pathway [3, 4]. Taxol biosynthesis is an intricate branch that involves at least 19 steps, from the diterpenoid precursor geranylgeranyl diphosphate (GGPP) to the final product [5]. The initial step is the cyclization of GGPP to taxadiene by a key enzyme,x taxadiene synthase (TS) [6]. Subsequently, the tricyclic taxane skeleton undergoes extremely complex modifications mediated by numerous oxygenases, acyltransferases, and benzoyltransferases [7]. Several key enzymes, including 2α-, 5α-, 7β-, 9α-, 10β-, and 13α-hydroxylases, taxadien-5α-ol *O*-acetyl transferase (TAT), and 10-deacetylbaccatin III 10-*O*-acetyltransferase (DBAT), were identified [3]. The last step is the synthesis of the functional taxol molecule by 3′-*N*-debenzoyl-2′-deoxytaxol-*N*-benzoyl transferase (DBTNBT) [8]. In order to uncover the mechanism underlying the transcriptional regulation of taxol biosynthesis, comprehensive understanding of the taxol biosynthetic pathway is vitally important. However, the complex and incompletely known biosynthetic pathway makes studies on its transcriptional regulation quite challenging.

To date, a great number of transcription factors (TFs) have been considered to play important roles in the regulation of various taxol pathway genes [4]. In the WRKY family, WRKY1, WRKY8, and WRKY47 in *Taxus chinensis* greatly enhanced the transcription levels of several taxol pathway genes [9, 10]. In the basic helix–loop–helix (bHLH) family, TcMYC2a in *T. chinensis* and JAMYC1, JAMYC2, and JAMYC4 in *Taxus cuspidata* were considered to function in the jasmonate-responsive expression of taxol biosynthesis pathway genes [11]. In the ERF family, a repressor, TcERF12, and an activator, TcERF15, affected taxol biosynthesis by recognizing the GCC-box on the promoter region of the *TS* gene [12]. Previous studies have revealed the functions of MYB members in the biosynthesis of different types of secondary metabolites [13]. Our recently published study identified an R2R3-MYB member in *Taxus media*, TmMYB3, which plays a potential role in the phloem-specific biosynthesis of taxol via enhancing the transcription of the *TBT* and *TS* genes [14].

In recent decades, sex-based differences in primary and secondary metabolisms of woody plants have been investigated [15]. The *Populus* genus is frequently used as a model woody plant to reveal the sexually dimorphic accumulation of metabolites. For example, the accumulation of primary metabolites was found to be sex-dependent in *Populus cathayana* leaves, while secondary metabolites accumulated sex-dependently in *P. cathayana* roots [16]. A previous proteomic analysis revealed sexual differences in the metabolic process of poplar trees under nitrogen deficiency [17]. Further study showed that male *Populus* trees had stronger capacity for carbon fixation and higher content of leaf abscisic acid and carbon sink than females under nitrogen deficiency [18]. In *Ginkgo biloba*, genomic analysis examined sex-related metabolic variations, such as specialized flavonoid metabolism and regulation [19].

More than 10 species in the *Taxus* genus have been identified by different groups, and taxoid accumulation varies greatly among these species. *Taxus* trees are dioecious. The question of whether accumulation of taxoids is sexually dimorphic has not been thoroughly investigated and the results of previous studies have remained contradictory. For example, no significant effect of sex on taxol content was observed in *Taxus wallichiana* trees [20]. However, in the bark of *Taxus baccata*, male trees yielded a higher level of taxol in comparison with female trees [21]. *Taxus media* is a natural hybrid species with great contents of taxoids, and no sex-specific foliar morphology is observed [14]. In *T. media*, whether a specific TF is involved in the transcriptional regulation of sex-dependent accumulation of taxol remains largely unclear.

In the present study, predominant accumulation of taxoids in female *T. media* trees was detected using a UPLC–MS/MS method. Furthermore, a female-specific TF, TmMYB39, was identified and its potential interaction with TmbHLH13 was identified. Our results may provide a possible explanation for the sex-dependent transcriptional regulation of taxol biosynthesis.

## Results

### Sexually dimorphic accumulation of taxoids in *Taxus media*

**Figure 1:**
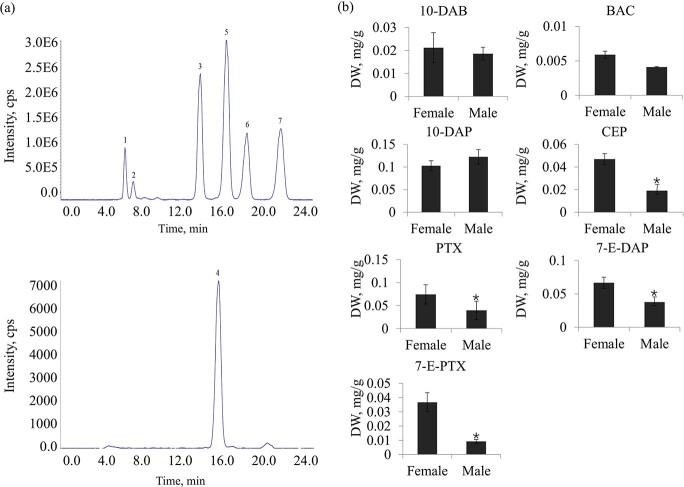
Determination of the taxoid contents in female and male *T. media* trees. **a** Representative TIC chromatograms of seven classic taxoids. **b** Levels of the seven taxoids were determined by a UPLC–MS/MS method. Peak 1, 10-DAB, 10-deacetylbaccatin III; peak 2, BAC, baccatin III; peak 3, 10-DAP, 10-deacetylpaclitaxel; peak 4, CEP, cephalomannine; peak 5, PTX, paclitaxel; peak 6, 7-E-DAP, 7-epi 10-desacetyl paclitaxel; peak 7, 7-E-PTX, 7-epipaclitaxel. ^*^P < .001.

Quantification analysis was carried out to explore the sexually dimorphic accumulation of taxoids in *T. media* trees. Seven well-known taxoids, including 10-deacetylbaccatin III (10-DAB), baccatin III (BAC), 10-deacetylpaclitaxel (10-DAP), cephalomannine (CEP), paclitaxel (PTX), 7-epi 10-desacetyl paclitaxel (7-E-DAP), and 7-epipaclitaxel (7-E-PTX), were determined, and total ion chromatography (TIC) chromatograms of these chemical compounds are showed in Fig. 1a. No significant differences in the accumulation levels of 10-DAB, BAC, and 10-DAP were observed between female and male trees. Interestingly, PTX accumulated to a significantly higher level in the female samples (0.07 mg/g) than male samples (0.04 mg/g). Besides, two derivatives of PTX, 7-E-DAP and 7-E-PTX, showed higher levels in female trees than male trees (Fig. 1b).

### Overview of the metabolomes

An untargeted metabolomic approach detected 4479 metabolites from 9445 ion features. Several parameters, such as TIC, *m/z* width, and retention time (RT) width, were analyzed to examine the quality of mass spectroscopy (MS) data. The TICs showed a high overlap of the data generated from all the samples (Supplementary Data Fig. S1). The analyses of *m/z* and RT widths showed that the metabolite extraction and sampling method reached the standards (Supplementary Data Fig. S2a and S2b). Based on their annotation information, an enormous number of potential metabolites were predicted and assigned to various KEGG (Kyoto Encyclopedia of Genes and Genomes) terms. The largest categories were ‘porphyrin and chlorophyll metabolism’ (26 metabolites), ‘diterpenoid biosynthesis’ (15 metabolites), ‘zeatin biosynthesis’ (10 metabolites), and ‘isoquinoline alkaloid biosynthesis’ (10 metabolites) (Supplementary Data Fig. 2c).

**Figure 2:**
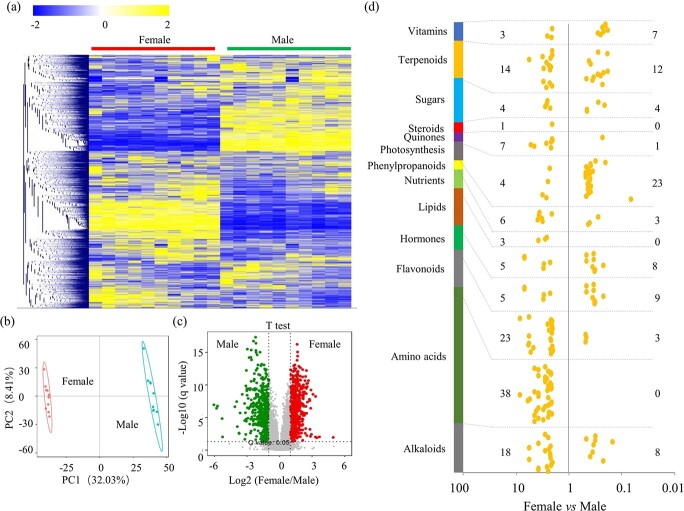
DAMs between female and male *T. media* trees. **a** Heat map of metabolite abundance in female and male trees (*n* = 10). The color scale ranges from −2 to +2 on a log_2_ scale. **b** PCA of metabolomes of female and male trees. **c** Volcano map of DAMs between female and male trees. **d** Analysis of DAMs grouped into different primary metabolic pathways. The numbers on the left indicate female-predominantly accumulated metabolites and the numbers on the right indicated male-predominantly metabolites. The scale ranges from 0.01 to 100.

### Analysis of differentially accumulated metabolites between female and male *Taxus media* trees

Metabolite profiling showed obvious variations in metabolomes between female and male *T. media* trees (Fig. 2a). Principal component analysis (PCA) showed that the PC1 and PC2 values were 32.03 and 8.41%, respectively, indicating a clear separation of the two groups and a good repeatability within the same group (Fig. 2b). Statistical analysis identified 1116 differentially accumulated metabolites (DAMs), including 623 female-predominantly accumulated metabolites and 493 male-predominantly accumulated metabolites (Fig. 2c).

Most of the DAMs were grouped into different metabolite biosynthesis pathways. For the terpenoid-related metabolites, 14 female- and 12 male-predominantly accumulated metabolites were identified; for the flavonoid-related metabolites, 23 female- and 3 male-predominantly accumulated metabolites were identified; for the amino acid-related metabolites, 38 female-predominantly accumulated metabolites were identified, and for the alkaloid-related metabolites, 18 female- and 8 male-predominantly accumulated metabolites were identified (Fig. 2d).

### Overview of transcriptomes

RNA sequencing yielded 44.25 Gb of raw data, including 21.6 Gb from the female samples and 22.65 Gb from the male samples (Supplementary Data Table S1). After filtration, the clean reads were assembled into 87 902 transcripts (N50 = 1786), with an average length of 664 bp, and 43 846 unigenes (N50 = 1792), with an average length of 498 bp (Supplementary Data Fig. S3a and S3b). The size distribution of the majority of the transcripts and unigenes was 200–500 bp, and only 13.89% of the unigenes and 15.35% of the transcripts were >2000 bp in length (Supplementary Data Fig. S3c). For functional annotation, 43 846 unigenes were annotated according to different databases (Supplementary Data Fig. S3d). The species annotation analysis suggested that the majority of *T. media* genes showed great similarities to *Picea sitchensis* (30.16%), *Amborella trichopoda* (10.62%), and *Nelumbo nucifera* (6.43%) (Supplementary Data Fig. S3e).

### Differentially expressed genes between female and male *Taxus media* trees

**Figure 3:**
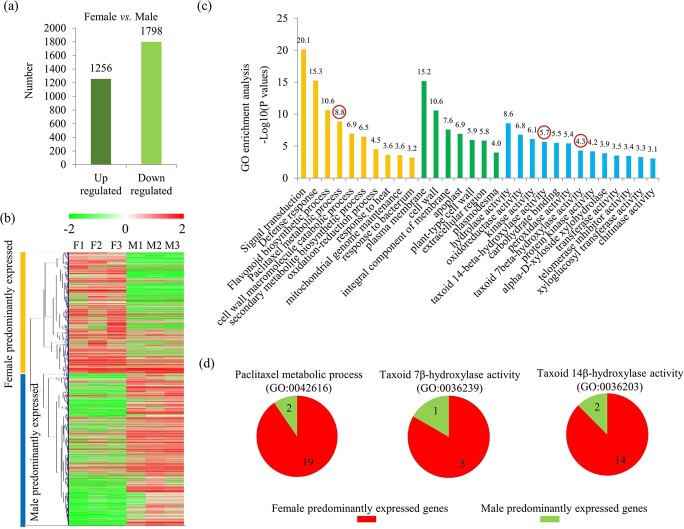
Analysis of DEGs between male and female *T. media* trees. **a** Numbers of up- and downregulated DEGs in female/male comparison. **b** Heat map of abundance of genes between female and male *T. media* trees. **c** GO enrichment analysis of DEGs between female and male *T. media* trees. Red circles indicate three taxol biosynthesis-related GO terms. **d** Numbers of female-predominantly and male-predominantly expressed genes associated with three taxol biosynthesis-related GO terms.

In total, 3054 differentially expressed genes (DEGs), including 1256 genes highly expressed in females and 1798 highly expressed in males, were identified (Fig. 3a). The gene expression profiles of *T. media* trees are shown in Fig. 3b. Detailed annotation information on the DEGs is listed in Supplementary Data Table S2. The DEGs were assigned to 164 significantly enriched gene ontology (GOs), among which three paclitaxel-related GOs, such as paclitaxel metabolic process (GO 0042616), taxoid 7β-hydroxylase activity (GO 0036239), and taxoid 14β-hydroxylase activity (GO 0036203), were included (Fig. 3c). Notably, all three paclitaxel-related GO terms mainly contained female-predominantly expressed genes (Fig. 3d).

### Integrated transcriptomic and metabolomic analyses of the taxol pathway

**Figure 4:**
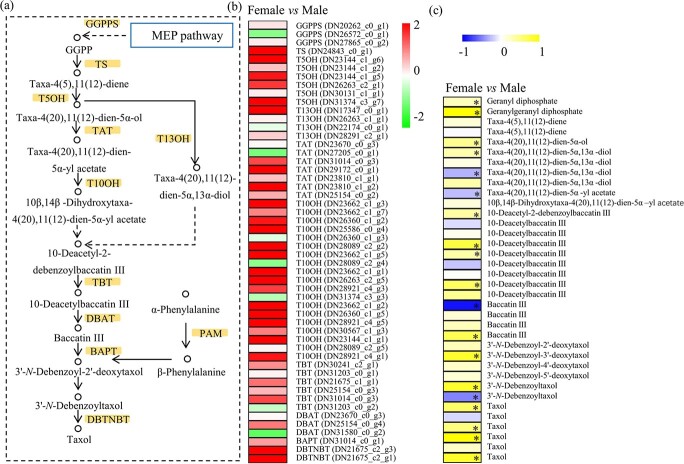
Integrated metabolomic and transcriptomic analyses for the taxol pathway. **a** Diagram of the taxol biosynthesis pathway. **b** Expression analysis of genes encoding enzymes related to the paclitaxel biosynthesis pathway between female and male *T. media* trees. The color scale ranges from −2 to +2 on a log_2_ scale. **c** Differential accumulation of several metabolites related to the taxol pathway between female and male *T. media* trees. The color scale ranges from −1 to +1 on a log_2_ scale.

A series of intermediate metabolites and enzymes have been reported to be involved in the sophisticated taxol pathway [5]. The biosynthesis outline from the MEP pathway to the final product, taxol, is shown in Fig. 4a. Based on the transcriptomes, 3 *GGPPS* genes, 1 *TS* gene, 6 *T5OH* genes, 4 *T13OH* genes, 7 *TAT* genes, 19 *T10OH* genes, 6 *TBT* genes, 3 *DBAT* genes, 1 *BAPT* gene, and 2 *DBTNBT* genes were identified. Sequences of these genes are listed in Supplementary Data Table S3. Most of these paclitaxel biosynthesis-related genes were highly expressed in the female trees (Fig. 4b). To verify their differential expression between male and female trees, seven paclitaxel biosynthesis-related genes were randomly selected and quantified using a quantitative real-time PCR (qRT–PCR) method. Our data showed that the expression levels of *GGPPS*, *TS*, *TBT*, *T13OH*, and *DBTNBT* genes were higher in female trees than male trees (Supplementary Data Fig. S4).

Based on the metabolomes, a large number of ion features referring to 12 taxol-related precursors or intermediates were detected. In detail, two precursors, GPP (one ion feature) and GGPP (one ion feature), greatly accumulated in female trees; for the intermediates, one feature referring to taxa-4 (20),11(12)-dien-5α-ol, one feature referring to taxa-4 (20),11(12)-dien-5α,13α-diol, one feature referring to 10-deacetyl-2-debenzoylbaccatin III, three features referring to 10-deacetylbaccatin, one feature referring to baccatin III, one feature referring to 3′-*N*-debenzoyl-3′-deoxytaxol, and one feature referring to 3′-*N*-debenzoyltaxol significantly accumulated in female trees; and for the end-product, four features referring to taxol significantly accumulated in female trees (Fig. 4c).

### Promoter sequences of taxol biosynthesis-related genes

Our previous study isolated five promoters of taxol biosynthesis-related genes, comprising *pTS* (DN26572_c0_g1), *pT7OH* (DN24804_c2_g5), *pT13OH* (DN22174_c0_g1), *pTBT* (DN31014_c0_g3), and *pDBTNBT* (DN21675_c2_g3) [14]. In the present study, four new paclitaxel biosynthesis-related promoters, comprising *pGGPPS* (DN26572_c0_g1), *pPAM* (DN25202_c0_g1), *pBAPT* (DN31014_c0_g1), and *pT10OH* (DN23144_c1_g1), were successfully cloned using a chromosome walking approach. The promoter sequences are listed in Supplementary Data Table S4. All nine promoter sequences were scanned for potential TF binding sites. Each of the promoter sequences of *pGGPPS*, *pPAM*, *pTBT*, *pT7OH*, and *pDBTNBT* contained one MYB-binding element (MBE), and each of the promoter sequences of *pTS*, *pT10OH*, and *pT13OH* contained two MBEs (Supplementary Data Fig. S4). Thus, we focused on MYB family TFs and their roles in sexually dimorphic accumulation of taxol.

### Basic analysis of the female-specific transcription factor TmMYB39

**Figure 5:**
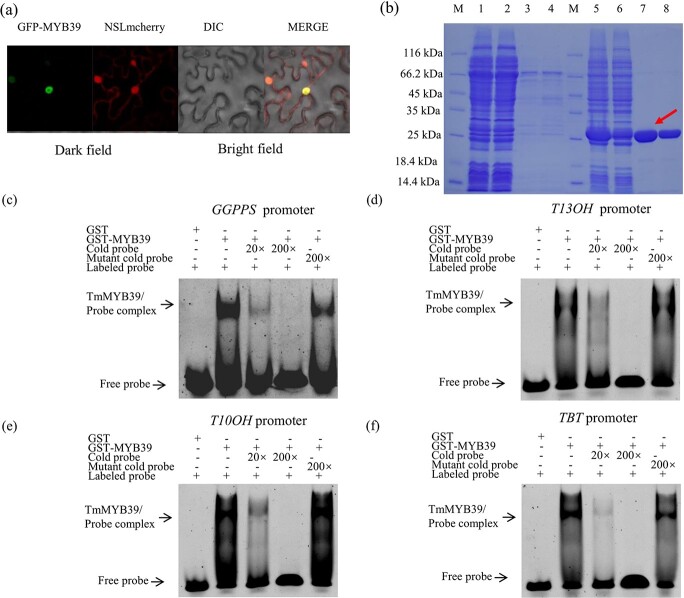
TmMYB39 binds to several taxol-related promoters. **a** Subcellular localization of TmMYB39. **b** Expression and affinity purification of the fused GST-TmMYB39 protein. M, protein marker; lanes 1–4, purification of GST–TmMYB39; lanes 5–8, purification of GST. Lanes 1 and 5, loading sample solutions; lanes 2 and 6, flow-through solutions; lanes 3 and 7, first elution with 10 mM GSH elution; lanes 4 and 8, second elution with 10 mM GSH. **c**–**f** Design of probes surrounding the MBEs in several taxol biosynthesis-related promoter regions. GST only or GST-TmMYB39 fusion protein was incubated with probes containing MBEs derived from the promoters of *GGPPS* (**c**), *T13OH* (**d**), *T10OH* (**e**), and *TBT* (**f**) genes, respectively. The symbols − and + represent absence and presence, respectively. ‘20×’ and ‘200×’ indicate the fold increases in probe amounts.

Based on the transcriptomes, 59 MYB family members were identified and the *TmMYB39* gene was significantly expressed in female *T. media* trees (Supplementary Data Table S5). The difference in expression of *TmMYB39* between female and male trees was confirmed by qRT–PCR (Supplementary Data Fig. S5). The full-length sequence of the *TmMYB39* gene was successfully isolated by PCR amplification. Further analysis revealed that TmMYB39 is a protein with 392 amino acid residues. Multiple sequence alignment analysis found a classic R2R3 domain at the *N*-terminus of TmMYB39 (Supplementary Data Fig. S6). The green fluorescent protein (GFP)-fused TmMYB39 was transiently expressed in tobacco leaves, suggesting that TmMYB39 was a nucleus-localized protein (Fig. 5a). To further investigate the function of TmMYB39, prokaryotic expression and affinity purification of the fused GST-TmMYB39 protein were performed (Fig. 5b).

### TmMYB39 binds to several taxol biosynthesis-related promoters

To uncover the role of TmMYB39 in the regulation of the taxol biosynthesis pathway, the binding of TmMYB39 to its downstream targets was determined. A series of probes surrounding the MBEs in the promoters of *GGPPS* (position −39/−15), *T10OH* (position −1047/−1023), *T13OH* (position −519/−495), and *TBT* (position −87/−63) were prepared (Supplementary Data Fig. S7). The electrophoretic mobility shift assay (EMSA) results indicated that TmMYB39 bound physically to the MBEs from the *GGPPS*, *T13OH*, *T10OH*, and *TBT* promoters. Adding competitive cold probes obviously weakened the binding of TmMYB39 to the downstream targets, while adding excess mutated probes had no effect on binding (Fig. 5c–f).

### TmMYB39 physically interacts with TmbHLH13

A number of MYB TFs play important roles in various biological aspects associated with bHLH proteins [22]. Based on the transcriptomes, 32 bHLH family members were annotated (Supplementary Data Table S5). A female-specifically expressed bHLH TF, *TmbHLH13*, encoding a putative protein with 687 amino acid residues, was identified. The difference in expression of *TmbHLH13* between female and male trees was confirmed by qRT–PCR (Supplementary Data Fig. S5). Multiple sequence alignment analysis showed that TmbHLH13 contained a classic bHLH structure similar to several well-studied *Arabidopsis* bHLH proteins (Supplementary Data Fig. S8).

**Figure 6:**
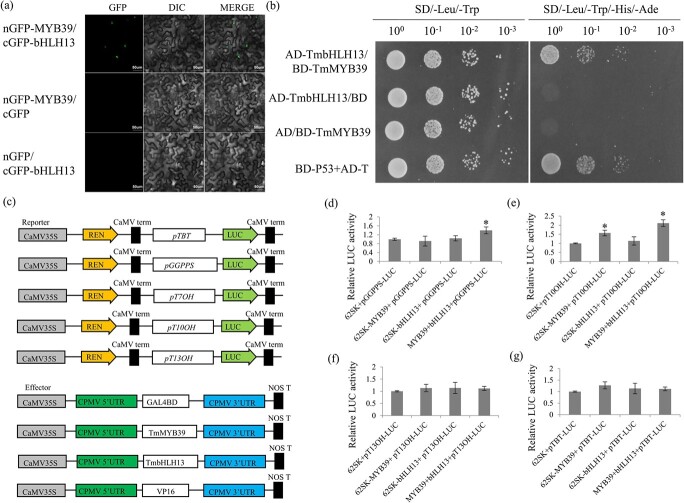
Functional analysis of two female-specific transcription factors, TmMYB39 and TmbHLH13. **a** BiFC analysis of the interaction between TmMYB39 and TmbHLH13. Fluorescence GFP signals indicate protein–protein interaction. Both of the nGFP-MYB39/cGFP and nGFP/cGFP-bHLH13 constructs were used as negative controls. **b** Y2H assay analysis of the interaction between TmMYB39 and TmbHLH13. Yeast cells transformed with AD-TmHLH13/BD and AD/BD-TmMYB39 constructs were used as negative controls, while those transformed with the BD-P53 + AD-T construct were used as positive control. **c** Overview of constructs prepared for dual-luciferase reporter assays. The promoter fragments of *TmTBT*, *TmGGPPS*, *TmT10OH*, and *TmT13OH* were ligated with the pGreenII 0800-LUC vector to produce the reporters. The effectors were produced by inserting the *TmMYB39* and *TmbHLH13* genes into the pGreenII-62-SK vector. **d**–**g** Effects of TmMYB39-TmbHLH13 complex on the luciferase activities of the GGPPS, T10OH, T13OH, and TBT reporters. Relative LUC activity represents the activity ratio of the firefly luciferase to *Renilla* luciferase. Each value is the mean ± standard deviation of three biological repeats. ^*^P < .05.

Bimolecular fluorescence complementation (BiFC) analysis and yeast two-hybrid (Y2H) assays were carried out to elucidate whether TmMYB39 interacts with TmbHLH13. Firstly, TmMYB39 was fused to GFP at the *N*-terminus and TmbHLH13 was fused to GFP at the C-terminus. Co-transformation of the two corresponding constructs produced a reconstituted functional GFP in the nucleus (Fig. 6a). Then, TmMYB39 fused with the BD of GAL4 and TmbHLH13 fused with the AD of GAL4 were created as a bait and a prey for Y2H assays, respectively. The results once again indicated the interaction between TmMYB39 and TmbHLH13 (Fig. 6b). After screening the promoter sequences of several taxol-related genes, no classic bHLH binding element was identified. Thus, we speculated that bHLH13 probably cannot bind to the promoters of downstream taxol-related targets by itself.

### Transcriptional activation of the *GGPPS* and *T10OH* genes by the TmMYB39-TmbHLH13 complex

The dual-luciferase reporter assay was carried out to investigate the function of TmbHLH13 in the MYB-bHLH complex. The full-length coding sequences of TmMYB39 (1176 bp) and TmbHLH13 (2061 bp) and the partial promoter sequences of the *GGPPS* (782 bp), *TBT* (1034 bp), *T10OH* (1394 bp), and *T13OH* (607 bp) genes were cloned and used in the present study (Fig. 6c). For the *GGPPS* promoter, TmMYB39, in the absence of bHLH13, could not activate *pGGPPS*, and co-expression of TmMYB39 and TmbHLH13 significantly upregulated the luciferase activity of p*GGPPS*. For the *T10OH* promoter, TmMYB39, in the absence of bHLH13, could slightly activate the expression of *pT10OH*, and co-expression of MYB39-bHLH13 could greatly activate the luciferase activity of the *pT10OH* reporter. Our data suggested that the *GGPPS* and *T10OH* genes could be transactivated by the TmMYB39-TmbHLH13 complex (Fig. 6d and e). However, the TmMYB39-TmbHLH13 complex had no significant effect on the luciferase activities of the *TBT* and *T13OH* reporters (Fig. 6f and g). Taken together, the results showed that the interaction between TmMYB39 and TmbHLH13 enhanced the promoter activities of the *GGPPS* and *T10OH* genes.

## Discussion

Sex-related differences in physiological and biochemical characteristics are universal in dioecious plants [23, 24]. In most cases, sexual dimorphism is explained as the consequence of opposite reproductive function. Additionally, sexual differences in responses to abiotic stresses as well as nutrient deficiencies have also been observed in seedlings before reproductive maturity [25]. However, studies on sex-specific accumulation of secondary metabolites are relatively scarce.

For years, *Taxus* plants have been the major natural materials for the extraction of taxol and other taxoids [26]. Distribution and accumulation levels of taxoids vary greatly among different species, tissues, and growth conditions [14, 27]. Our study revealed the sexually dimorphic accumulation of taxoids and other metabolites in *T. media* trees. Predominant accumulation of taxoids in female *T. media* suggests that female trees may be a more valuable source for the extraction of taxol. Flavonoids are another important class of bioactive substances and play essential roles in plant adaptation to the terrestrial environment [28]. Interestingly, flavonoid-related metabolites predominantly accumulated in female *T. media* trees, suggesting stronger responses of female trees to environmental stresses.

The taxol biosynthesis pathway consists of many functional genes and regulators [29]. To date, most of the functional genes involved in the taxol biosynthesis pathway have been identified [30]. Recently the search for efficient regulators of taxol biosynthesis-related genes, especially transcriptional regulators, has gained a lot of attention [31]. Analysis of jasmonic acid-responsive transcriptomes identified a number of TFs involved in taxol biosynthesis, including WRKYs, JAZs, and ERFs [9, 12]. In our study, a comprehensive transcriptomic analysis was performed, identifying 3054 DEGs between female and male *T. media* trees. Among these DEGs, a number of TFs were included, providing massive sequence information for screening novel transcriptional regulators.

Several functional genes are regarded as having been identified as the downstream targets of the taxol biosynthesis-related TFs. In *T. chinensis*, ERF12 and ERF15 participate in the expression regulation of the *TS* gene by binding to a GCC-box from −150 to −131 bp [12]. Overexpression of *T. chinensis* MYC2a upregulates the expression levels of the *TS*, *TAT*, and *DBTNBT* genes through binding to the G-box and E-box [11]. TcWRKY1 enhances *DBAT* expression in *T. chinensis* suspension cells by interacting with two W-box (TGAC) *cis*-elements [10]. In our study, 1 *TS* gene, 3 *TAT* genes, 4 *T5OH* genes, 2 *DBTNBT* genes, and 13 *T10OH* genes were significantly expressed in the female trees, suggesting their potential roles in sexually dimorphic accumulation of taxoids. For example, T5OH, T10OH, and T13OH are important oxygenation enzymes involved in taxadiene hydroxylations [7]. In female *T. media* trees, higher expression levels of these hydroxylase encoding genes might lead to significant accumulation of taxoid intermediates. The last acylation in the taxol biosynthetic pathway is catalyzed by DBTNBT [32]. Predominantly expressed *DBTNBT* in female trees may contribute to the female-predominant accumulation of taxol. However, the TFs involved in the sexually specific accumulation of taxoids are largely unknown.

Here, the function of TmMYB39 in the sexually dimorphic accumulation of taxol was analyzed. According to the number of MYB domains, MYB TFs can be grouped into four subclasses, R1, R2, R3, and R4 [33]. Multiple sequence alignment analysis identified two R repeats in the *N*-terminus of TmMYB39, suggesting that TmMYB39 is a typical R2R3-type MYB TF. The homologous gene of TmMYB39 in *Arabidopsis thaliana* is AtMYB013, which is predominantly detected in the shoot apex zone [34]. However, homologous genes may display functional diversity and differential expression patterns in the process of evolution.

Several MYB-related binding elements have been reported in model plants. For example, MRE1 (AMCWAMC) and MRE2 (GGWTW) are identified as classic MYB binding elements in *A. thaliana* [35]. Based on previously published elements, eight taxol-related promoter sequences containing 11 MBEs were selected as potential downstream targets of TmMYB39. Four promoters of three taxol-related genes could be bound to TmMYB39, suggesting that the binding of TmMYB39 to its targets was dependent on the surrounding sequences of MBEs.

R2R3-type MYB TFs that regulate secondary metabolic processes exist widely in medicinal plants [36]. In *Bacopa monnieri*, MYB35 is involved in the biosynthesis of monoterpene alkaloids by binding to the promoter of the *BmG10H-1* gene [37]. In *Salvia miltiorrhiza*, SmMYB1 enhances the biosynthesis of phenolic acid by increasing the expression of *CYP98A14*, *CHI*, and *ANS* genes [38]. In *Glycyrrhiza uralensis* Fisch., two R2R3-MYB TFs, GlMYB4 and GlMYB88, positively regulate the biosynthesis of flavonoids in licorice cells [39]. Our previous study has identified an R2R3-type TmMYB3 that is involved in the phloem-specific accumulation of taxol by regulating the expression of the *TBT* and *TS* genes [14]. As another MYB family member, TmMYB39 was considered to participate in sex-specific accumulation of taxol.

In the present study, results from the EMSA assay indicated four potential downstream target genes of TmMYB39. It is acknowledged that MYB interacts with bHLH, mostly together with WD40 repeat protein, forming a complex to regulate multiple biological processes [40]. Different functions of the MYB-bHLH complex have been uncovered in various plants, such as the MdMYB308L-MdbHLH33 complex in apple cold tolerance, the AtMYB4-TT8 complex in *Arabidopsis* flavonoid biosynthesis, and WP1-TT8 in *Medicago truncatula* carotenoid-derived flower pigmentation [22, 41, 42]. In our study, results from the BiFC and Y2H assays indicated the physical interaction between TmMYB39 and TmbHLH13; therefore TmMYB39 might play an essential role in taxol biosynthesis through an ‘MYB-bHLH’ module. *Arabidopsis* has evolved various MYB-bHLH-WD40 complexes for anthocyanin and proanthocyanidin pigment regulation. After many years of investigating the regulation mechanism of flavonoid biosynthesis, the MYB-bHLH-WD40 complex is considered to be predominantly required for the terminal steps of the flavonoid biosynthetic pathway [43]. Besides taxoids, flavonoids represent another major class of bioactive compounds in *Taxus* [44, 45]. The MYB39-bHLH13 complex may function not only in taxol biosynthesis, but also in other secondary metabolic pathways, such as flavonoid synthesis, which needs to be explored in future studies.

**Figure 7:**
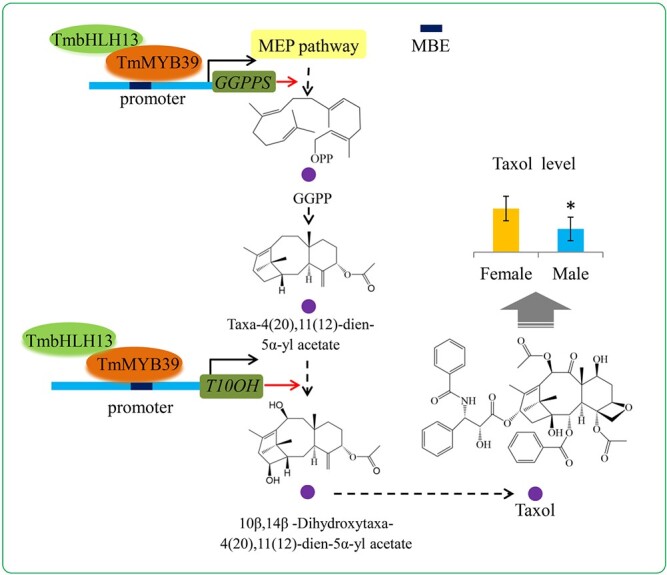
A hypothetical model for the function of the TmMYB39-TmbHLH13 complex in the transcriptional regulation of the taxol biosynthesis pathway. The female-specific TmMYB39-TmbHLH13 complex has a significant role in the sexually dimorphic biosynthesis of taxol in *T. media* trees.

Among the four potential target genes, expressions of the *GGPPS* and *T10OH* genes were significantly increased by the TmMYB39-TmbHLH13 complex. GGPP is produced via the MEP pathway, and serves as one of the major precursors for the taxane skeleton [46]. In *Taxus* trees, cyclization of GGPP to taxa-4(5),11(12)-diene was reported to be a key step in the taxol biosynthesis pathway [47]. In plants, GGPP is synthesized by GGPP synthetase (GGPPS), which can use DMAPP, GPP, and FPP as substrates [48]. In our study, the TmMYB39-TmbHLH13 complex transactivated the expression of *GGPPS*, indicating a more sufficient precursor supply in female trees. T10OH is responsible for one step of the serial hydroxylation reactions of taxa-4 (20),11(12)-dien-5α-yl acetate, which can produce an important taxoid intermediate in the taxol biosynthesis pathway [7]. Female-predominant expression of *TmMYB39* and *TmbHLH13* and interaction between TmMYB39 and TmbHLH13, together with transcriptional activation of *GGPPS* and *T10OH* by the TmMYB39-TmbHLH13 complex, may build a possible model for the regulation of the sexually dimorphic accumulation of taxol (Fig. 7).

In conclusion, predominant accumulation of taxoids was observed in female *T. media* trees, suggesting that female trees might have more value for large-scale cultivation. An integrated transcriptomic and metabolomic analysis identified a great number of DEGs and DAMs, revealing comprehensive differences between female and male *T. media* trees. Moreover, a female-specifically expressed TmMYB39 and its interaction protein (TmbHLH13) were identified. The TmMYB39-TmbHLH13 complex might play an important role in taxol biosynthesis by transactivating the transcription of the *GGPPS* and *T10OH* genes. Our data provide a potential explanation for the sexually dimorphic biosynthesis of taxol in *T. media* trees.

## Materials and methods

### Plant materials

Five-year-old *T. media* trees were grown in a greenhouse within the Cangqian campus of Hangzhou Normal University, Hangzhou, China. Independent twigs from female and male trees were harvested for metabolite extraction (25 mg each, *n* = 10) and RNA sequencing (25 mg each, *n* = 3).

### Determination of taxoid contents

Fresh twigs were collected from six female and male *T. media* plants. The preparation of crude extracts was carried out according to a previously published method [49]. The test taxoids included 10-DAB, BAC, 10-DAP, CEP, PTX, 7-E-DAP, and 7-E-PTX. Separation and quantification of the seven taxoids were performed according to our previously described method [49]. Six independent repeats were used in our study.

### Transcriptomic profiling

Total RNA isolation was carried out using a RNeasy Mini Kit (Qiagen, Hilden, Germany). The mRNAs were purified and cut into small fragments. The fragmented mRNAs were used to produce cDNA libraries using a library preparation kit (Illumina, San Diego, USA). RNA sequencing was processed on an Illumina 4000 platform (LC-Bio, Hangzhou, China) to produce raw reads according to the manufacturer’s protocol.

High-quality clean reads were used for *de novo* assembly of the transcriptomes of *T. media* using the Trinity assembly program [50]. For unigene annotation, the longest sequence representing each unigene was BLASTed against different databases.

### Bioinformatics of transcriptomic datasets

For expression level calculation, the clean reads were mapped onto the reference sequence using the Bowtie program (ver. 0.12.7). After normalization, the transcription levels of unigenes were determined by the RPKMR method. The DEGs between female and male trees were analyzed using the DESeq R program (ver. 1.10.1). The absolute values of log_2_(female/male) were calculated and applied to draw a heat map using MeV ver. 4.9.0.

Most of the DEGs were classified into different GO and/or KEGG metabolic pathways by the Blast2GO program (https://www.blast2go.com/). For the enrichment analysis, a GO or KEGG group with a corrected *P* value <.05 was considered to be significant.

### Real-time PCR analysis

Total RNAs extracted for the RNA sequencing were used in RT–PCR validation. The RT–PCR experiment was performed according to our previous study [49]. The primer sequences used for the RT–PCR are listed in Supplementary Data Table S6.

### Untargeted metabolomic profiling

Metabolite sampling was processed according to a published method [51]. After drying and resuspension in 50% methanol, the extracts were separated using a Waters ultraperformance liquid chromatography (UPLC) system (Hertfordshire, UK) with a BEH C18 column (100 × 2.1 mm, 1.7-μm particle size). Then, the resulting metabolites were analysis using an HPLC–MS/MS system. Details of the mobile phase and gradient elution conditions were the same as those in previously published work [52].

The metabolites from the reversed-phase separation column were determined using a high-resolution MS/MS TripleTOF 5600 Plus System (Sciex, UK) in both positive and negative modes. The parameters of Q-TOF operation, including curtain gas pressure, ion-source gas pressure, interface heater temperature, ion-spray voltages, MS mode, TOF mass range, total cycle time, and dynamic exclusion during acquisition, were set as in previously published work [52].

### Analysis of untargeted metabolomic data

The resulting MS/MS features, including peak picking, peak grouping, and peak annotation, were obtained using XCMS software. Metabolites were annotated using the online KEGG and PLANTCYC databases. Detailed information on metabolite annotation is listed in Supplementary Data Table S7. An in-house program, metaX, was used to preprocess the intensity of data. Further bioinformatic analysis of the untargeted metabolomic dataset was performed according to published work [52].

### Promoter isolation and gene cloning

*T. media* genome DNA was extracted from fresh twigs. The promoter regions of the *GGPPS*, *PAM*, *TS*, *TBT*, *BAPT*, *T13OH*, and *DBTNBT* genes were obtained using a Clontech GenomeWalker Kit (TaKaRa, Dalian, China). The genome DNA was firstly digested by different blunt-end restriction enzymes, and then the resulting samples were ligated with adaptors from the GenomeWalker Kit. The adaptor-added DNA fragments were used as templates for two rounds of nested PCR.

Partial *TmMYB39* and *TmbHLH13* sequences were obtained from the *T. media* transcriptome. The full-length sequences of TmMYB39 and TmbHLH13 were cloned by PCR amplification. The sequences of primers used for the PCR amplification are shown in Supplementary Data Table S6.

### Subcellular localization and phylogenetic analysis

The cDNA sequences of *TmMYB39* and *TmbHLH13* were inserted into the pH7FWG2.0 vector integrated with GFP. NSLmcherry-RFP was used as a marker. All primer sequences are shown in Supplementary Data Table S6. All constructions were transformed into *Nicotiana tabacum* epidermal cells using a GV3101-mediated transient expression system.

### Yeast two-hybrid and bimolecular fluorescence complementation system

The Clontech Two Hybrid System (Dalian, China) was used to check the interaction between TmMYB39 and TmbHLH13. The coding sequences of TmMYB39 and TmbHLH13 were fused to BD-GAL4 in pGBKT7 vector and AD-GAL4 in pGADT7 vector, respectively, with EcoRI/BamHI restriction sites. The resulting constructs were co-transformed into yeast cells (AH109). Co-transformation of pGBKT7 and pGADT7 vectors was used as negative control. Transformed yeast cells were spread on plates containing SD/−2 or SD/−4 to detect interactions.

### Prokaryotic expression and electrophoretic mobility shift assay

The cDNA of the *TmMYB39* gene was cloned into pGEX-4 T and transformed into *Escherichia coli* cells (DE3) to get recombinant TmMYB39-His tag protein. The recombinant protein was firstly induced by 1 mM IPTG and then purified using His60 Ni Superflow Resin (Clontech) according to the manufacturer’s method. TmMYB39-His tag fusion protein were isolated by 12% SDS–PAGE.

EMSA was carried out as described in our previously published work [14]. Two reported MYB-related *cis*-elements, CAGTTA and TGGTTA, were used to design probes. Sequences containing MYB-specific elements derived from the promoters of *GGPPS*, *PAM*, *TS*, *TBT*, *BAPT*, *T13OH*, and *DBTNBT* were labeled with 5′6-FAM fluorescent dye. Unlabeled probes were treated as competition probes and probes with sequence changed into CCCGGG were treated as mutation probes. All probe sequences are listed in Supplementary Data Table S6. EMSA was performed using the Light Shift Chemiluminescent EMSA Kit (GS009, Beyotime, China) according to the manufacturer’s instructions.

### Dual-luciferase reporter assay

The pGreenII0800-LUC and pGreenII62-SK vectors were used. The cDNAs of TmMYB39 and TmbHLH13 were inserted into the pBD vector as effectors. Internal *Renilla luciferase* (REN) was treated as reporter. The promoters of *GGPPS*, *PAM*, *TS*, *TBT*, *BAPT*, *T13OH*, and *DBTNBT* were inserted into the pGreenII 0800-LUC double-reporter vector, respectively. The resulting constructs were co-transformed into tobacco leaf cells using *Agrobacterium tumefaciens* GV3101 strain. Luciferase (LUC) activities were calculated using a dual-luciferase assay kit (Promega). The primer sequences are shown in Supplementary Data Table S6.

### Statistical analysis

The Wilcoxon text was carried out to identify DAMs between the female and male sample groups. The supervised partial least-squares discriminant method was applied to determine the variables between the female and male sample groups. False discovery rate analysis was applied to identify DEGs between the female and male sample groups. A *P* value of <.05 adjusted using the Benjamini–Hochberg method was used as a threshold.

## Supplementary Material

Web_Material_uhac062

## Data Availability

The metabolomic datasets generated and analyzed during the current study are available in the Baidu Netdisk (https://pan.baidu.com/s/1dC0gRo_mNwLXaqhrP6oNVQ) with extraction code nclg. The transcriptomes of *T. media* trees have been uploaded to the NCBI database under accession number GSE175645.
