# Supplementary material for: Role of female-predominant MYB39-bHLH13 complex in sexually dimorphic accumulation of taxol in Taxus media
Source: Hortic Res. 2022 Mar 14;9:uhac062. doi: 10.1093/hr/uhac062 (PMC9233167; doi:10.1093/hr/uhac062)
Supplement: Web_Material_uhac062 [file web_material_uhac062.zip › HR_SI_Figures.pdf]

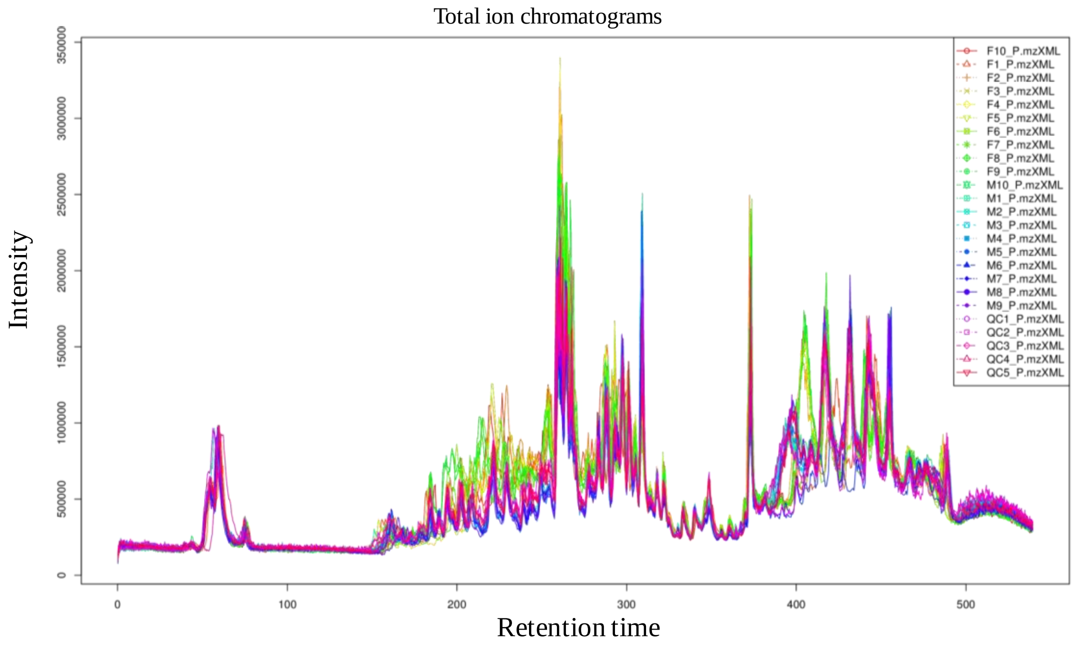

Figure S1 **TIC chromatograms of the metabolomes of the female and male *T. media* trees.**

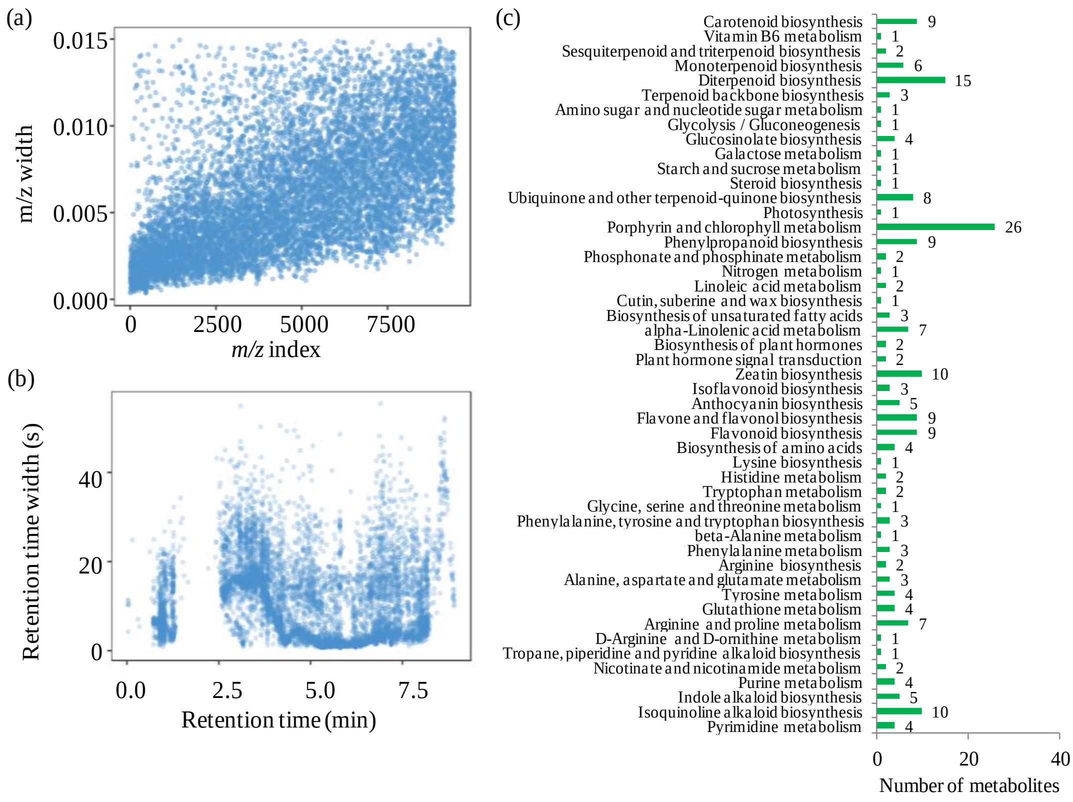

Figure S2 **Overview of the metabolomes of the female and male *T. media* trees.** Quality control parameters, including *m/z* widths (a) and retention time widths (b) of the metabolomes. (c) Analysis of the DAMs belonging to different major metabolic categories. Numbers of the DAMs belonging to different KEGG categories.

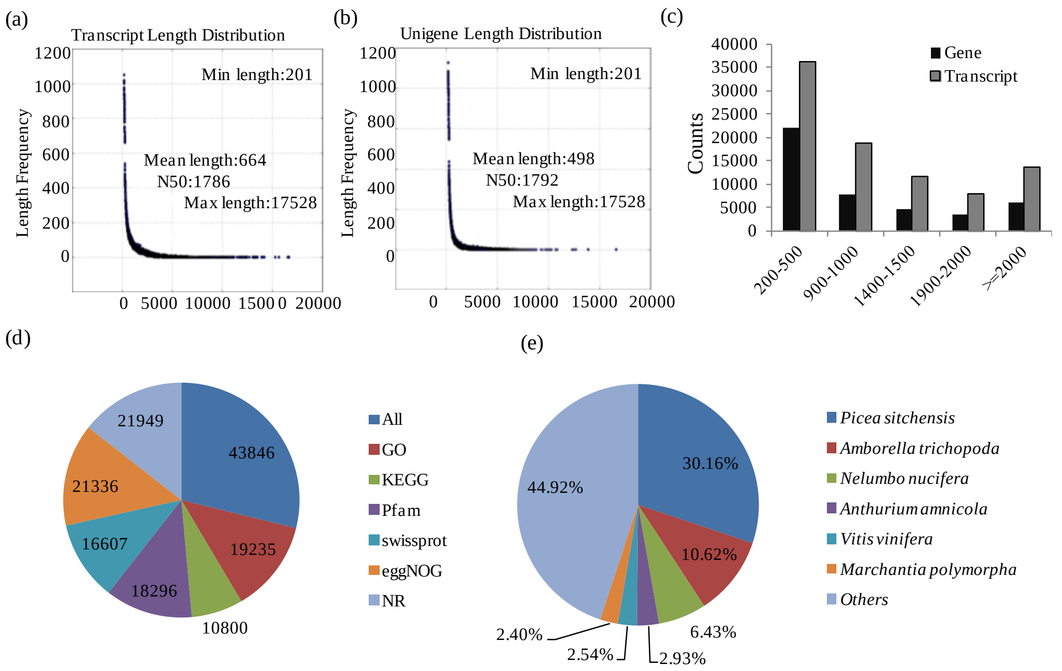

Figure S3 **Overview of the transcriptomes of the female and male *T. media* trees.** (a) The detailed information of assembled transcripts. (b) The detailed information of assembled unigenes. (c) The length distribution of assembled transcripts and unigenes of the female and male *T. media* trees. (d) The number of unigenes annotated by different databases, including NR, Swissprot, eggNOG, KEGG, GO and Pfam. (e) Species distribution of all annotated unigenes.

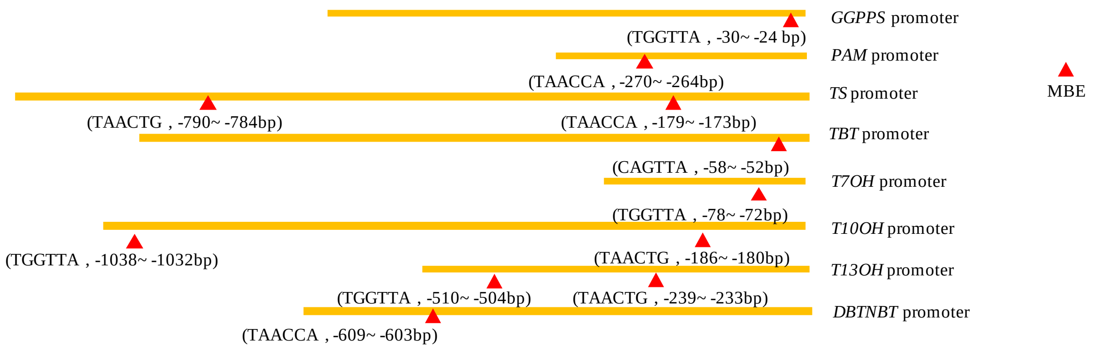

Figure S4 **Screening of MBEs in the promoter regions of several taxol biosynthesis-related genes. Crimson triangles indicated the MBEs.**

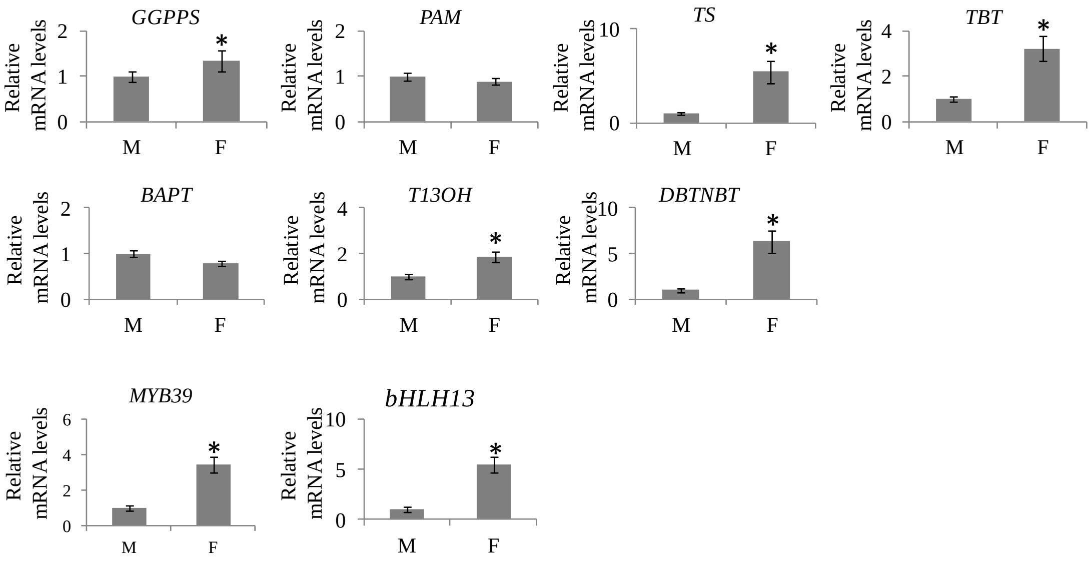

Figure S5 **Analysis of genes involved in the paclitaxel biosynthesis pathway.** Expression analysis of seven key genes, including *GGPPS*, *PAM*, *TS*, *TBT*, *BAPT*, *T13OH*, *DBTNBT*, *MYB39*, and *bHLH13* in the male and female *T. media* trees. Each value is the mean ± SE of three biological repeats. '*' represents significant differences ($P < 0.05$).

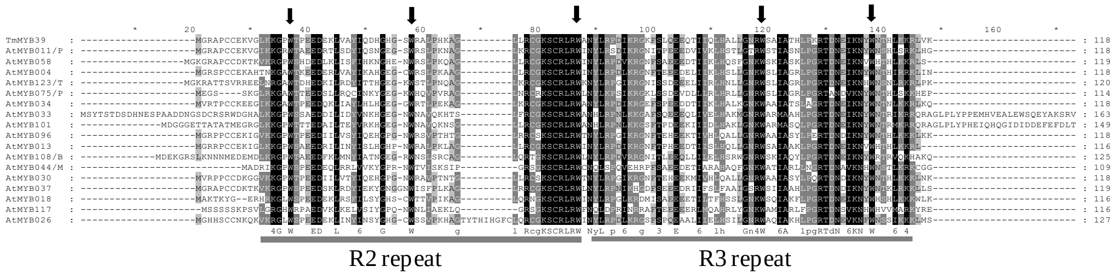

Figure S6 **Multiple sequence alignment analysis of TmMYB39 and several MYB TFs from *Arabidopsis thaliana*.** Conserved amino acid residues are highlighted with a black background and a gray background indicates partial conservation of amino acid residues. The conserved tryptophans (W) are indicated by arrows.

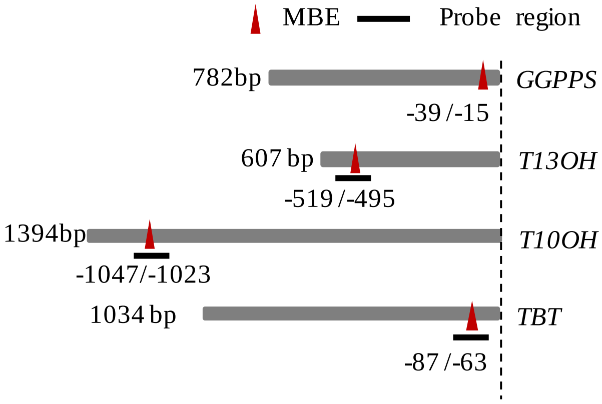

Figure S7 **The detial information of MBE in the promoters of *GGPPS*, *T13OH*, *T10OH* and *TBT* genes.**

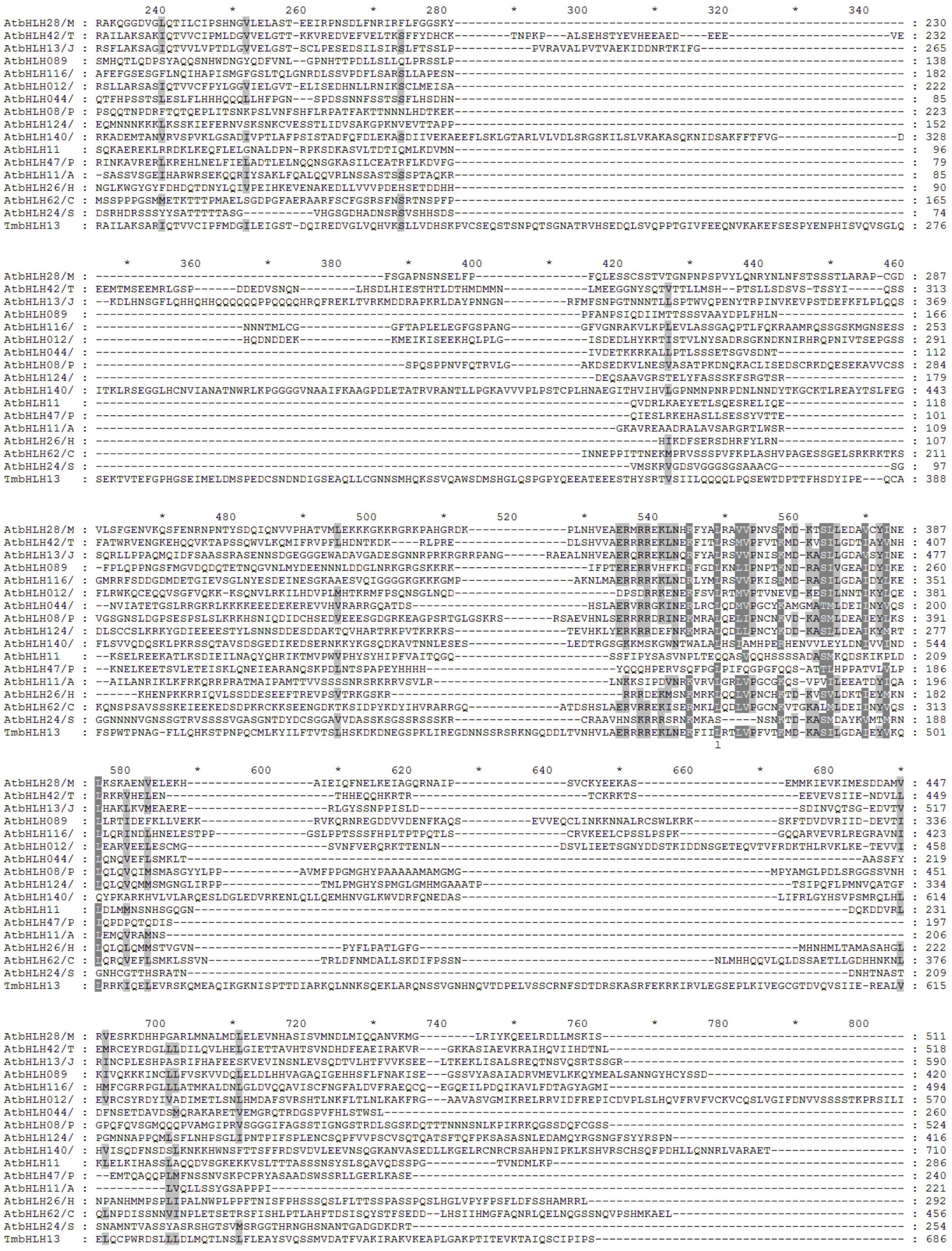

Figure S8 **Multiple sequence alignment analysis of the bHLH family proteins.**
